# Use of the Reversible Myogenic to Lipogenic Transdifferentiation Switch for the Design of Pre-clinical Drug Screening in Idiopathic Pulmonary Fibrosis

**DOI:** 10.3389/fbioe.2020.569865

**Published:** 2020-09-15

**Authors:** Arun Lingampally, Matthew R. Jones, Shirisha Bagari, Chengshui Chen, Stefano Rivetti, Saverio Bellusci

**Affiliations:** ^1^Key Laboratory of Interventional Pulmonology of Zhejiang Province, Department of Pulmonary and Critical Care Medicine, First Affiliated Hospital of Wenzhou Medical University, Wenzhou, China; ^2^Department of Pulmonary and Critical Care Medicine and Infectious Diseases, Universities of Giessen and Marburg Lung Center (UGMLC), Member of the German Center for Lung Research (DZL), Cardio-Pulmonary Institute, Justus-Liebig University Giessen, Giessen, Germany

**Keywords:** idiopathic pulmonary fibrosis, aging, *in vitro* models, alveolar type 2 cells, activated myofibroblast, lipofibroblast

## Abstract

Idiopathic Pulmonary Fibrosis (IPF) is an end-stage lung disease characterized by excessive extracellular matrix (ECM) deposition from activated myofibroblasts (MYFs) and tissue scarring. Eventually leading to stiffening of the lung, capable of assuming only limited gas exchange function. So far two drugs, pirfenidone [acting via TGF-β (transforming growth factor beta) inhibition] and nintedanib (a pan-tyrosine kinase receptor inhibitor) have been approved for IPF patients. They both act on the activated MYF by reducing the expression of fibrotic markers. Unfortunately, these drugs are only slowing down fibrosis formation and as such do not represent a cure for this lethal, devastating disease. We previously reported that activated MYF originate, at least in part, from lung fibroblast resident cells called lipofibroblasts (LIF). During resolution, these activated MYF can transdifferentiate into LIF. We propose that this reversible myogenic/lipogenic transdifferentiation switch paradigm can be used to screen for drugs capable of triggering the lipogenic differentiation of activated MYFs. Ideally, these drugs should also induce the reduction of pro-fibrotic markers alpha smooth muscle actin2 (ACTA2) and collagen 1A1 (COL1A1) in activated MYF and as such would represent important alternatives to the approved drugs. The goal of this review is to summarize the current knowledge and limitations of the current strategies aiming to carry out methodical pre-clinical drug screening in pertinent *in vitro*, *ex vivo*, and *in vivo* models of IPF. These models include (1) *in vitro* culture of primary fibroblasts from IPF patients, (2) *ex vivo* culture of precision cut lung slices from end-stage IPF lungs obtained from transplant patients, and (3) bleomycin-induced fibrosis mouse models in the context of lineage tracing of activated MYF during resolution. For all these assays, we propose the innovative use of lipogenic read outs for the LIFs.

## Idiopathic Pulmonary Fibrosis (IPF)

IPF is a fatal lung disease with unknown ethology. It is a pathological condition characterized by excessive extracellular matrix (ECM) deposition and tissue scarring, progressively leading to stiffening of the lung, respiratory paralysis, and death. IPF patients are often diagnosed at end stage, and the mean survival rate post-diagnosis is 3 years. The average annual incidence of pulmonary fibrosis is 17.4 per 100,000 persons worldwide (El Agha et al., 2017a; Zhao et al., 2020). It is widely postulated that IPF starts with repetitive injury and chronic inflammation of the alveolar epithelium. Injured epithelial cells regulate secretion of profibrotic and inflammatory mediators like matrix metalloproteins (MMP), growth factors, cytokines and chemokines (Selman and Pardo, 2006; Mora et al., 2017), which consequently results in the recruitment and chemo-attraction of activated myofibroblasts (MYFs) in the fibrotic precursor region known as fibrotic foci. Fibrotic foci are enriched with the profibrotic mediator transforming growth factor beta (TGF-β), which induces the transition of resident lung fibroblasts to MYFs. During homeostasis, TGF-β is in an inactive form bound to the ECM. However, during fibrosis, TGF-β becomes activated in the ECM and is also secreted by the alveolar epithelium (Wipff et al., 2007; Fernandez and Eickelberg, 2012; Zhao et al., 2020).

Activated MYFs are the core drivers for IPF progression and ECM deposition. Activated MYFs express alpha smooth muscle actin2 (ACTA2), and are characterized by apoptotic resistance and excessive ECM production. Activated MYFs have been proposed to arise through transdifferentiation from different precursors during fibrosis. Recent studies have shown that the activated MYFs arise from pericytes (Marriott et al., 2014), from CD45^+^ COL1^+^ CXCR4^+^ bone marrow-derived circulating fibrocytes (Phillips et al., 2004) and from lipofibroblasts (LIFs) (El Agha et al., 2017b). In addition, it has been proposed that alveolar epithelial type 2 (AT2) cells could also be a source of activated MYF through a process of epithelial-to-mesenchymal transition (EMT) (Kim et al., 2006). However, this is controversial as lineage tracing of AT2 cells in mice in the context of bleomycin-induced pulmonary fibrosis does not show any contribution of AT2 cells to the newly formed myofibroblasts foci (Rock et al., 2011). Given all these potential sources of activated MYFs, it is likely that these cells constitute a quite heterogeneous population which so far has not been well-characterized. It is also likely that subpopulations in this heterogenous activated MYF population may respond differently to drugs (Kheirollahi et al., 2019).

It is therefore important to clearly define the types of read outs that will be implemented in the context of pre-clinical drug screening models in idiopathic pulmonary fibrosis. Our proposed models focus on the transdifferentiation of the MYFs into an important mesenchymal cell type, the LIFs. LIFs are proposed to be critical for the maintenance of AT2 stem cells and as such, are critical for lung repair after injury. The AT2-LIF interaction will be further developed in the next chapter.

## Interaction Between Alveolar Epithelial Type 2 (AT2) Stem Cells and Lipofibroblasts (LIFs)

The alveolar epithelium consists of alveolar type 1 (AT1) and AT2 cells. AT1 cells are instrumental for gas exchange and AT2 cells secrete surfactant. At birth, pulmonary surfactant is crucial for reducing the surface tension in the alveoli thereby allowing gases exchange and for adapting to the transition from 3 to 21% oxygen (Torday, 2003; Torday et al., 2003). Recent discoveries suggest that a subpopulation of AT2 has a higher capacity for self-renewal, and the ability to differentiate to AT1 cells during homeostasis and injury (Barkauskas et al., 2013; Zacharias et al., 2018). Recent studies from Nan Tang et al. have shown that mechanical forces contribute to differentiation of the AT2 to AT1 cells during lung development and pneumonectomy (PNX)-induced alveolar regeneration. They also observed that Cdc-42 (Cell division cycle 42), a member of the RhoGTPase family, is required for differentiation of AT2 cells to AT1 cells. Ablation of *Cdc-42* in AT2 cells exhibited impaired regeneration and elevated mechanical stress, which led to the activation of latent Tgf-β. Activated Tgf-β signaling in turn led to the initiation and progression of fibrosis from the periphery to the center of the lung (Liu et al., 2016; Li et al., 2018; Wu et al., 2020). Interestingly, AT2 senescence, by activating senescence-associated secretory phenotype (SASP), and loss of AT2 cells due to persistent injury as well as altered AT2 self-renewal capacity are observed in IPF patients. AT2 cells are therefore active regulators of fibrosis. As they collaterally get damaged, this leads to the induction of the fibrotic phenotype, which includes the activation of resident fibroblast by profibrotic factors such as TGF-β (Yao et al., 2019; Parimon et al., 2020).

## LIFs Significantly Contribute to the Pool of Activated MYFs During Fibrosis

As of today, the characteristics of the different types of resident fibroblasts in the adult lung are still unclear. During the early 1970s, two distinct types of interstitial cells were observed in neonatal rat lungs, namely, the lipid interstitial cell (aka LIF) and the non-lipid interstitial cell (O’Hare and Sheridan, 1970; Vaccaro and Brody, 1978). Lipofibroblasts are lipid droplet containing fibroblasts, anatomically located in close proximity to AT2 cells. These cells are Acta2-negative and also express Adipose differentiation related protein (*Adrp*)/Perilipin2 (*Plin2*). LIFs emerge during the late pseudoglandular stage of mouse lung development. Studies involving genetic tracing using the Fibroblast growth factor 10 (*Fgf10*^*icre*^) mouse model as well as mouse mutants displaying impaired Fgf10/Fibroblast growth factor receptor 2b (Fgfr2b) signaling demonstrated the embryonic origin of LIFs and the role of Fgf10 in LIF differentiation during lung development (El Agha et al., 2014; Al Alam et al., 2015). Ablating a single allele of *Fgf10*, or its receptor *Fgfr2b*, in the mesenchyme of lungs exhibited significant decrease in LIF formation at birth. Adipocytes and LIFs share similarities in development. Fgf10 is the principal regulator of adipogenesis, Fgf10 signaling activates peroxisome proliferator activated receptor gamma (*Pparg*) expression via retinoblastoma protein and CCAAT-enhancer-binding protein alpha (pRb and C/EBPα) complex. *Fgf10* deficient mice exhibited impaired differentiation of the white adipose tissue (Sakaue et al., 2002). In addition, Fgf10 appears to also control the proliferation of the white adipose tissue (Konishi et al., 2006).

LIF synthesizes and secretes Fgf10, which acts in both a paracrine manner on the epithelium for branching morphogenesis and differentiation, and in an autocrine fashion to control the differentiation of LIF progenitors toward the lipogenic lineage (Bellusci et al., 1997; Al Alam et al., 2015; Jones et al., 2019). In the adult lung, during homeostasis and repair, LIFs contribute to the AT2 stem cell niche; LIFs and AT2 co-cultured in Matrigel formed the so-called “alveolospheres” in a 3D organoid model (Barkauskas et al., 2013). These alveolospheres are the consequence of the self-renewal of the AT2 stem cell as well as their differentiation toward AT1 cells. Interestingly, to date, surface markers specific for LIFs have not yet been discovered. Several dyes for Lipid droplets have been described such as Sudan III stains red to yellowish–red, Oil Red O stains red to reddish–orange and Sudan Black B stains black. However, LipidTOX dye appeared to be dye of choice for the assays described within this review. LipidTOX dye stains neutral lipid droplets with a remarkably high affinity and can be used to follow the formation and differentiation of cells within the lipogenic lineage. The detection of the dye via fluorescence allows the use of flow cytometry to quantify and isolate live LIFs during development and repair.

**FIGURE 1:**
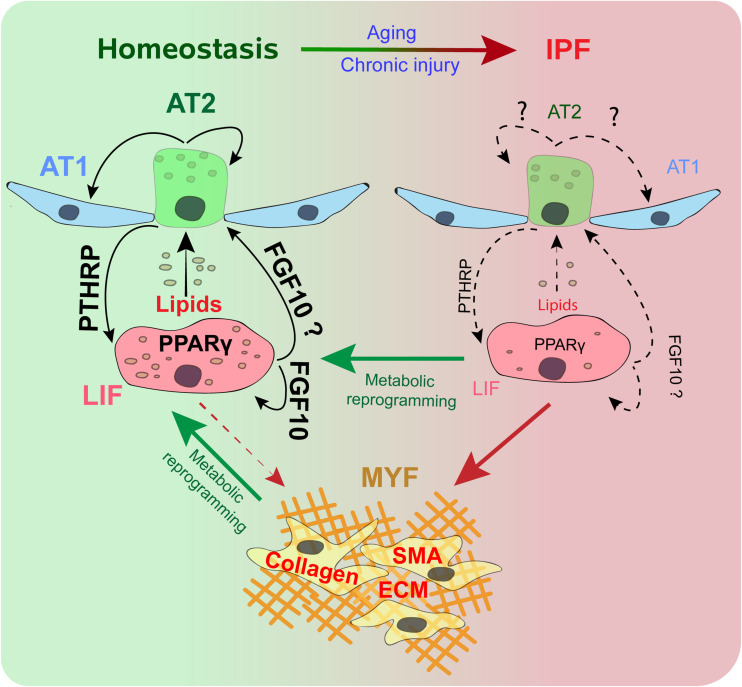
Alveolar type 2 (AT2) and lipofibroblast (LIF) interaction during homeostasis and IPF. During homeostasis, a subset of AT2 cells helps in the renewal and maintenance of the AT2 and alveolar type 1 (AT1) pools. The PTHRP/PPARγ axis is vital for lipid trafficking and surfactant production. FGF10 signaling maintains LIFs and acts as s stem cell niche for AT2. During IPF, AT2 cells senesce. Disruption of the PTHRP/PPARγ axis and FGF10 leads to loss of LIFs. LIFs differentiate to smooth muscle actin (SMA) positive Myofibroblasts (MYF), with excessive extracellular matrix (ECM) deposition. Metabolic reprograming promotes the dedifferentiation of MYFs to LIFs, and helps retain the LIF population.

Recent studies in mice demonstrated that LIFs significantly contribute to the pool of activated MYFs during fibrosis. Using *Adrp*^*Cre–ERT2*^ mice, a LIF genetic tracing model, it was shown that LIFs transition to an activated MYF phenotype in bleomycin-challenged mice. Surprisingly, during fibrosis resolution, a beneficial process which is happening in young mice (but unfortunately not in humans), lineage-labeled activated MYFs, labeled using the *Tg(Acta2-CreERT2); tomato^flox^* mice, do not undergo apoptotic clearance, but instead acquire a lipogenic program to transdifferentiate back to LIFs (El Agha et al., 2017b). This reversible transdifferentiation switch from LIF to activated MYF opens the way for the testing of drugs capable of stimulating the transition of the activated MYF toward the LIF phenotype (Figure 1).

## Metabolic Reprograming During Fibrosis

The survival of a living organism is tightly associated with energy metabolism. ATP, the product of metabolism, is vital for survival and used in various cellular pathways. Metabolic dysregulation contributes to organ fibrosis through a decrease in mitochondrial biogenesis (Bueno et al., 2020). Mitochondrial biogenesis is crucial for cell survival and energy production by cellular respiration. AT2 cells in bleomycin-induced fibrosis display swollen mitochondria, a phenomenon which leads to apoptosis of the AT2 cells. A recent study indicated that thyroid hormone T3 and T4 (metabolic regulators) attenuated bleomycin-induced fibrosis in a mouse model. This antifibrotic effect was due to normalization of the mitochondrial structure and mitochondrial function, thereby inhibiting AT2 apoptosis (Yu et al., 2018). Recent studies have shown that 5’ adenosine monophosphate-activated protein kinase (AMPK) activity is essential for mitochondrial biogenesis and fibrosis resolution. Reduced AMPK activation is observed in MYFs during fibrosis, leading to the accumulation of apoptotic resistant proliferative MYFs. This pathological fibrotic state is attenuated by AMPK activators, like 5-aminoimidazole-4-carboxamide ribonucleotide (AICAR) and metformin, by sensitizing MYFs to apoptosis and inhibiting their proliferation (Rangarajan et al., 2018). In brief, altered metabolism is associated with increased prevalence and incidents of fibrosis. AT2 cell regeneration and apoptotic clearance of profibrotic MYFs are essential for IPF therapy.

During homeostatic conditions, resident fibroblasts express ECM at physiological rates. The ECM regulates tissue function, maintains the 3D-structure, as well as orchestrates the repair process. During pathological states, resident fibroblasts, and the recruited fibrocytes, increase ECM deposition in the fibrotic regions. ECM is composed of fibronectin, laminin, collagens, proteoglycans, and glycosaminoglycans. Collagen is the major protein of the ECM and collagen type 1 is predominantly deposited during fibrosis. Collagen is synthesized in the endoplasmic reticulum as pre-pro-collagen and pro-collagen, then packed and secreted into the extracellular space by the Golgi apparatus. Pro-collagen is cleaved and cross-linked to form insoluble collagen fibers (Zhao et al., 2020). Collagen is mostly composed of the amino acids proline, lysine and glycine. Glycine which contributes to one third of collagen, is synthesized *de novo* by the serine-glycine one carbon pathway, a pathway that is catalyzed by the Phosphoglycerate Dehydrogenase (PHGDH) enzyme. PHGDH expression is elevated in the fibrotic foci regions and Inhibition of the PHGDH attenuated TGF-β-mediated collagen synthesis in fibrosis (Nigdelioglu et al., 2016; Hamanaka et al., 2018). In another study, Endo et al. (2003) demonstrated an increase in arginase, an enzyme which catalyzes arginine to ornithine, the precursor for proline and hydroxyproline, which contributes to ECM remodeling in bleomycin-challenged mice. Lysyl oxidases (LOX) catalyze and facilitate crosslinking of collagen and elastin by aldehyde groups, thereby inhibiting ECM degradation. Interestingly, hypoxia-inducible transcription factor (HIF) regulates LOX during hypoxia. LOX represses *E-cadherin* expression, causing EMT (Schietke et al., 2010). Inhibition or knock-down of LOX showed decreased recruitment of MYFs in bleomycin-challenged mice (Cheng et al., 2014). Glycolysis and ATP are upregulated in MYFs during fibrosis, which contributes to collagen synthesis. Fatty acid oxidation contributes to collagen degradation. Both collagen synthesis and degradation should be at physiological rates during homeostasis (Rabinowitz and Mutlu, 2019). ECM collagen unwinds and is cleaved by MMPs in the extracellular space. The cleaved collagen fibers, via fatty acid translocase CD36 (aka platelet glycoprotein 4), translocate into fibroblasts for lysosomal degradation (Rabinowitz and Mutlu, 2019; Zhao et al., 2020). CD36 activates PPARγ, the master regulator of lipogenesis (Febbraio et al., 2001). Tgf-β upregulation during fibrosis inhibits CD36, leading to dysregulating in collagen degradation. Therefore the extensive screening of drugs capable of correcting different types of metabolic dysregulations is likely to be a very promising venue for fibrosis therapy (Zhao et al., 2020).

In a recent study, it was reported that the first line antidiabetic and metabolic drug metformin, acting as a AMPK-activator, was capable to enhance the activated MYF to LIF transition both (1) *in vitro* in primary fibroblasts from IPF patients, (2) *ex vivo* in precision cut lung slices from end-stage IPF lungs obtained from transplant patients, and (3) *in vivo* using bleomycin-induced fibrosis mouse models in the context of lineage tracing of activated MYF during resolution (Kheirollahi et al., 2019). These three types of assay constitute the base for the pre-clinical drug screening in idiopathic pulmonary fibrosis.

## Assay 1: Use of Primary Fibroblasts From Human Lungs

Due to their ability to replicate human physiology, *in vitro* human cell-based models have been extensively used in drug testing during toxicological and pharmacological studies. Human lung fibroblast cultures were established during the early 1960s in the Wistar Institute (Hayflick and Moorhead, 1961). Later, the fibroblasts were immortalized by simian virus 40 (SV40). However, cell immortalization resulted in permanent phenotypic changes and may no longer reflect *in vitro* their normal counterpart (Jensen et al., 1963).

To circumvent this problem, primary human lung fibroblast culture from end stage IPF or Donor human lungs (usually obtained due to the need for lung transplantation) is now the standard *in vitro* model to identify the key underlying mechanisms of IPF, as well as for pre-clinical drug testing. It is critical to mention here that due to pathological variations between IPF lungs, it is important to validate, prior to drug screening, on at least 10 different primary IPF cultures that the selected cells respond (by decreasing the expression of fibrotic markers *ACTA2, COL1A1*) to currently approved drugs commonly used in IPF such as pirfenidone and nintedanib.

In this assay, it is sometimes difficult to observe differences between IPF and Donor fibroblasts in terms of ACTA2 and collagen 1A1 (COL1A1) expression. For this reason, drug screening often includes a pre-treatment with recombinant TGF-β to activates the IPF fibroblasts and enhance the expression of the profibrotic markers ACTA2, COL1A1, and Fibronectin. Recent studies illustrated that the antidiabetic drugs like metformin and rosiglitazone attenuate the fibrotic phenotype in this *in vitro* IPF fibroblast model (El Agha et al., 2017b; Rangarajan et al., 2018; Kheirollahi et al., 2019). Metformin is an anti-hyperglycemic agent used as a first line drug in type 2 diabetes treatment. Metformin has a dual, antifibrotic and lipogenic, effect. Metformin inhibits the TGF-β1 mediated ECM proteins expression via AMPK (pT172-AMPK). The AMPK agonist AICAR inhibits ECM proteins via inducing autophagy and mTOR-dependent ribosomal S6 phosphorylation. Metformin induces also lipid droplet formation as well as increase in *PLIN2* and *PPARg* expression. Metformin induced the expression and secretion of bone morphogenetic protein 2 (BMP2). Activation of the BMP2 signaling axis in activated MYFs led to the phosphorylation of PPARγ at serine-112, which in turn led to the induction of *PLIN2* expression and lipid droplet formation. Inhibition of BMP2, along with treatment with metformin, abolishes lipid droplet formation and *PLIN2* expression. However, AMPK-dependent COL1A1 inhibition was not altered in these studies.

Interestingly, compared to metformin, pirfenidone and nintedanib displayed quite a modest effect on reduction of the expression of fibrotic markers in primary IPF culture (Kheirollahi et al., 2019). This could be due to drug resistance in IPF patients upon long-term treatment and justifies the efforts for fast-tracking the discovery of new drugs.

**FIGURE 2:**
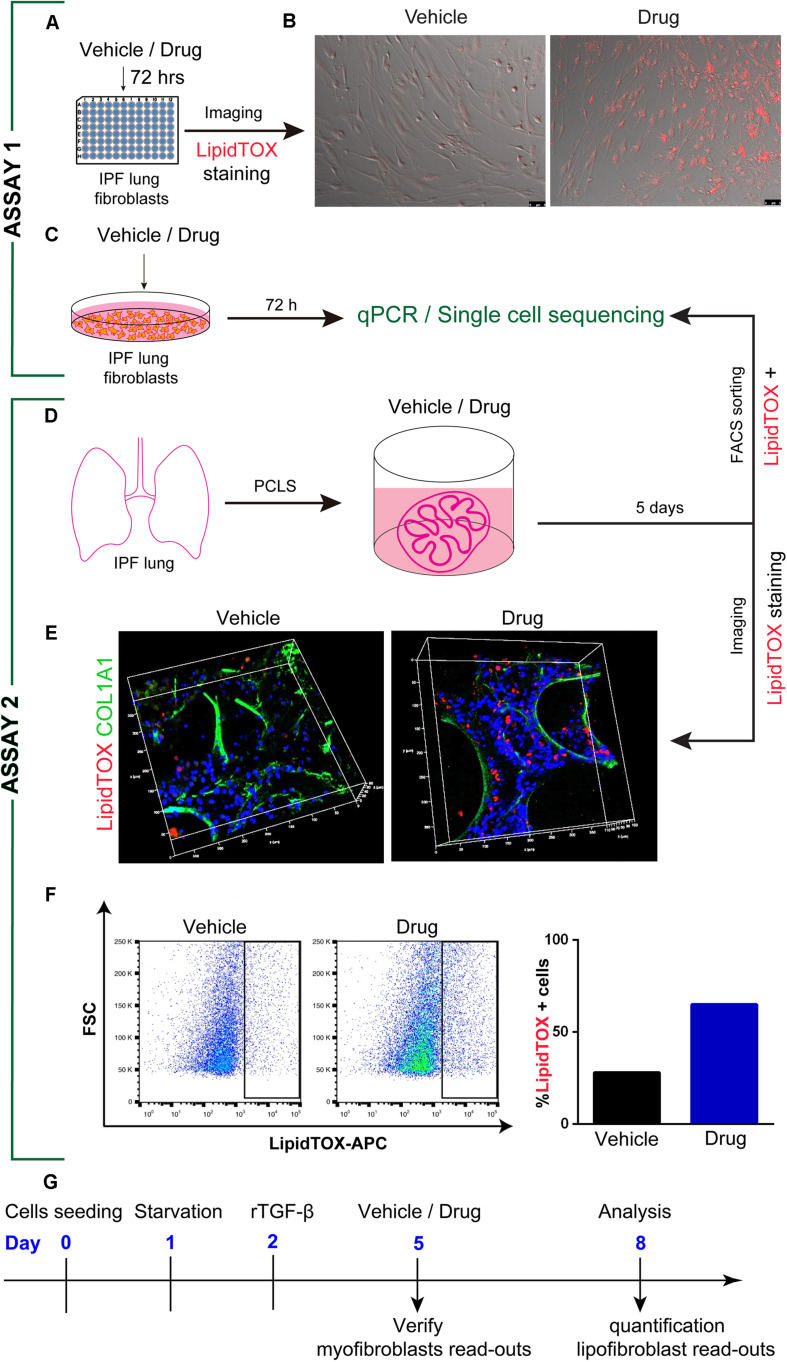
*In vitro* drug screening models in idiopathic pulmonary fibrosis. **(A)** Experimental set-up, where primary IPF lung fibroblasts are treated with drugs or vehicle in a 96 well plate. **(B)** Time-lapse imaging with presence of LipidTOX dye to visualize lipid drops for 72 h. **(C)** Treatment of primary IPF lung fibroblasts with drugs or vehicle for 72 h and gene analysis by qPCR or single-cell sequencing. **(D)** PCLS preparation from fresh IPF lung explants and treatment of PCLS with drugs or vehicle for 5 days, FACS-sorting of LipidTOX positive cells, and analysis of genes by qPCR or single-cell sequencing. **(E)** Confocal images of PCLS after 5 days treatment and staining with LipidTOX dye and anti-Collagen1 antibody. **(F)** FACS-based analysis and quantification for vehicle and drug treated PCLS or IPF lung fibroblasts. **(G)** Timeline for assay 1 (Figure 2 adapted from Kheirollahi et al., 2019). Scale bars: **(B)** 5 μm; **(E)** 400 μm, 400 μm, 100 μm (XYZ-axis).

Rosiglitazone, a PPARγ agonist and antidiabetic drug, antagonizes the TGF-β-mediated fibrotic phenotype. Time-lapse imaging of primary human lung fibroblasts with antifibrotic drugs (metformin/rosiglitazone) and LipidTOX dye is a novel model to visualize in real-time the transition from a profibrotic to an antifibrotic phenotype. This assay can be amenable to high throughput screening by adapting the culture of IPF fibroblasts to 96 or 384 wells plates and incorporating fluorescent imaging techniques or fluorescence quantification of each well through automated systems allowing the quantification of LipidTOX staining (Figure 2) or the total fluorescence signal through a proper cell lysis. However, in this later case, it is important to use a lysis method that will not degrade the lipids. In addition, drugs capable of inducing the lipogenic differentiation should then be validated by qPCR for their capacity to reduce *COL1A1* and *ACTA2* expression.

Another important aspect to consider in the screening of drugs using the assay 1 is the possibility to have false positives. To overcome this possibility, our MYF to LIF screen includes both negative and positive internal controls. The assay described is based on the relative quantification of the LIF phenotype (treated vs. non-treated, the later serving as a negative internal control for each IPF primary culture). This is important as a high variability between human primary samples treated with drugs *in vitro* has been described (Kheirollahi et al., 2019). In addition, for each primary culture of IPF, we will compare the results obtained with the new drugs to the ones previously obtained with metformin (which will provide another independent positive internal control). Finally, these new drugs will also be evaluated independently for their capacity to reduce the expression of profibrotic markers. It will be therefore very unlikely that we are going to select drugs based on false positive effect on the MYF to LIF primary screen.

## Assay 2: Use of Ex-Vivo Precision Cut Lung Slices

Currently, the precision-cut lung slices (PCLS) technique has gained increasing attention as an *ex vivo* human disease model. Historically, Warburg et al. (1927) introduced the usage of tissue slices culture in cancer research. The development of the microtome enabled rapid production of slices and minimized the stress on the tissues (Krumdieck et al., 1980). It is important to mention here that the lung transplant used to generate PCLS should be delivered from the surgery room to the processing facility as quickly as possible (usually within 12 h). This requires strong coordination to efficiently carry out isolation of fibroblasts, vibratome sectioning for PCLS as well as isolation of epithelial, mesenchymal, endothelial and immune cells by flow cytometry. In addition, while it is possible to cryopreserve live cells such as primary fibroblasts, this technique is not efficient to preserve the integrity of PCLS, which therefore require fresh lungs. Initially, PCLS were used to study proximal airways in toxicological studies, and were later optimized for the distal lung tissues (Placke, 1987; Kheirollahi et al., 2019). Human lung tissues enable a more sophisticated analysis of fibrosis mechanisms and provide material for pre-clinical drug testing. PCLS are thought to preserve human physiology and lung functionality, by which interactions between cells can be maintained during fibrosis and drug testing. Use of PCLS as a secondary assay for the validation of drugs capable of inducing lipogenic differentiation (see Assay 1) is a natural and optimal follow up in the overall pre-clinical validation strategy. Due to the use of pertinent human material, PCLS reduces the challenges in the interpretation and limitations which are commonly observed in animal fibrosis models (see assay 3).

It was recently shown that metformin has antifibrotic properties in the PCLS model. In this study, 200 μm PCLS display preserved tissue architecture for up to 5 days in culture. Human IPF PCLS treated with metformin for 5 days exhibited macroscopically a relaxed structure with open spaces. In the future, it would be important to incorporate optical/physical techniques allowing to quantify this relaxed state. Metformin treatment increased the number of LipidTOX stained cells with a corresponding decrease in collagen deposition. Such a decrease in collagen, together with other fibrotic specific proteins, as well as the induction of lipofibroblast characteristic markers (e.g., PLIN2 and PPARγ), could also be validated by western blot using the whole PCLS. Quantification by flow cytometry indicated increase in LipidTOX positive cells in the PCLS treated with metformin vs. vehicle suggesting a transition from a MYF to LIF phenotype in IPF lungs. Analysis of histological images supported that metformin attenuated fibrosis, and tissue sections confirmed the relaxed structure and open airspaces associated with decreased collagen deposition. Time-lapse imaging of PCLS with antifibrotic drugs and LipidTOX dye was a novel model used to visualize in real-time the transition from a profibrotic to an antifibrotic phenotype. As IPF lungs may not be easily available in every institution, this assay should be kept as a second level validation of the drugs identified using the primary culture of IPF fibroblasts and LipidTOX staining as a main read out.

Due to the limitation in obtaining fresh lungs, it is important to consider alternative approach. One of them is the use of 3D Matrigel-based lung organoids developed *in vitro* from human pluripotent stem cells (hPSCs). Recently, Snoeck et al. have developed a model to generate lung bud organoids (LBOs) form hPSCs. These LBOs contain pulmonary endoderm and mesoderm. LBOs cultured in Matrigel displayed the formation of branching airways and alveolar structures. This model was carried out to study the lung fibrosis associated with Hermansky–Pudlak syndrome (HPS). Deletion in hPSCs of *HPS1*, *HPS2* and *HPS4* followed by the generation of corresponding LBOs showed elevated fibrotic markers (Chen et al., 2017; Strikoudis et al., 2019). New experimental models using LBOs combined with CRISPR/Cas9 gene editing could be used as a therapeutic and drug screening model in the coming future.

## How Are Assay 1 and 2 Advancing IPF Research?

The first advantage of these two assays is that human materials coming from patients with IPF are being used. This allows testing of new drugs directly on primary human cells relevant for the disease. The drawback of Assay 1 is that it is lacking the *in vivo* environment. In addition, the phenotype of the cells may change due to *in vitro* culture and in our experience, there is quite a lot of variability in the response to drugs between different sets of primary IPF fibroblast (reflecting the heterogeneity of the human population). In addition, as these IPF fibroblasts are from patients who have been treated with Pirfenidone and/or Nintedanib, the response of the IPF cells from these patients is somehow limited due likely to resistance to these drugs (Kheirollahi et al., 2019). As aforementioned, to overcome the variability between IPF fibroblasts, a pre-treatment with recombinant TGF-β is required to activate the IPF fibroblasts and enhance the expression of the profibrotic markers ACTA2, COL1A1, and Fibronectin. Furthermore, to have statistical reliable results, it is recommended to use around 10 independent primary IPF cell fibroblasts. Assay 1 is mostly testing the efficacy of new drugs in the context of resistance to the currently available drugs. It may therefore be important to treat the cells with a combination of the old drugs with the new ones under investigation to find a potential synergistic effect. The same limitations are also applicable for the assay 2, even though PCLSs represent a much better model due to the preservation of the complex cellular environment. As aforementioned, LIFs and AT2 cells interactions are crucial for the maintenance of the AT2 stem cells during homeostasis. Loss of LIFs in IPF correlates with AT2 cells injury and loss. Therefore, the analysis of new drugs using PCLS (Assay 2) should allow monitoring and quantifying the increase in LIF formation and the collateral impact on AT2 cells proliferation and differentiation. In addition, it will be important to quantify the effect of these new drugs on the restoration of the native lung structure by the clearance of the deposited collagen.

## Assay 3: Use of Murine Fibrosis Models

This is the last arm of the screening strategy that we propose for drugs first identified in primary culture of IPF (assay 1) and then validated using PCLS (assay 2). Murine models are widely accepted and used to study pulmonary fibrosis, due to their ability to represent the native complex biological system for understanding disease pathogenesis. Currently, various murine fibrosis models exist, including bleomycin, amiodarone, silica, asbestosis, cytokine overexpression, fluorescent isothiocyanate, and radiation-induced (Tashiro et al., 2017). Ideally and when possible, the models described above should also integrate the lineage tracing of the activated MYF [using the *Tg(Acta-CreERT2); Tomato^flox^* mice] (El Agha et al., 2017b). This would allow an easy monitoring of the activated MYF during resolution.

However, murine models have limitations. One limitation is that young mice, capable of fibrosis resolution are often used. IPF occurs usually in old men and is unfortunately not reversible (Garcia, 2011). It would therefore be more appropriate to use older mice (around 12–18 months of age) instead of the 2–3 months old mice used in most of the studies. Aging is associated with a natural decline in physiological and cellular processes. The hallmarks of the aging process are cellular senescence, epigenetic changes, stem cell depletion, genomic mutations, mitochondrial dysfunction, metabolic changes, and altered cellular communications and interactions (Sgalla et al., 2018; Bueno et al., 2020). Aging leads to significant prevalence of chronic lung diseases like COPD and IPF. An age and sex dimorphism study revealed aged male mice are more susceptible to fibrosis than young males, and, interestingly, young and old female mice showed less fibrosis than male mice (Redente et al., 2011). In addition, most of these model do not integrate the progressive nature of the disease, which in humans takes decades to develop. Interestingly, such models mimicking the progressive injury to AT2 cells with mutation in *surfactant protein C* (*Sftpc)* leading to endoplasmic reticulum stress (Lawson et al., 2011) or the recently described mice displaying *Cdc-42* deletion in AT2 cells (Wu et al., 2020) are now available. If drugs can work in the context of these mouse models, then there is a higher chance that these drugs will be more efficient in humans. Prior to the initiation of clinical trials, these mouse models should definitively be included for the final selection of drugs potentially efficient in IPF.

## Conclusion

The reversible myogenic/lipogenic switch was used as an example to illustrate different pre-clinical models. We have detailed a three arms strategy for the high flow throughput selection and subsequent validation of new drugs potentially used for the treatment of IPF patients. These drugs are selected based on their lipogenic differentiation potential as well as on their antifibrotic potential. The reversible myogenic/lipogenic switch between LIF and activated MYF is a new paradigm that has not been used so far in the context of drug screening. Finding drugs displaying this dual lipogenic/antifibrotic activity will allow restoring the functionality of the LIFs, an essential component of the AT2 stem cell niche, thereby promoting enhanced lung repair.

## Author Contributions

AL wrote the manuscript. MJ edited the manuscript. SBa made the illustrations. CC, SR, and SBe proposed the concept for the manuscript, guided the writing process, and made the final corrections. All authors contributed to the article and approved the submitted version.

## Conflict of Interest

The authors declare that the research was conducted in the absence of any commercial or financial relationships that could be construed as a potential conflict of interest.
